# Ranking and filtering of neuropathology features in the machine learning evaluation of dementia studies

**DOI:** 10.1111/bpa.13247

**Published:** 2024-02-19

**Authors:** Mohammed D. Rajab, Teruka Taketa, Stephen B. Wharton, Dennis Wang, Cognitive Function and Ageing Neuropathology Study, and for the Alzheimer's Disease Neuroimaging Initiative

**Affiliations:** ^1^ Sheffield Institute for Translational Neuroscience University of Sheffield Sheffield UK; ^2^ Department of Computer Science University of Sheffield Sheffield UK; ^3^ Singapore Institute Clinical Sciences Agency for Science Technology and Research (A*STAR) Singapore Singapore; ^4^ Bioinformatics Institute Agency for Science Technology and Research (A*STAR) Singapore Singapore; ^5^ National Heart and Lung Institute Imperial College London London UK

**Keywords:** Alzheimer's disease, collinearity, dementia, feature selection, machine learning, neuropathology

## Abstract

Early diagnosis of dementia diseases, such as Alzheimer's disease, is difficult because of the time and resources needed to perform neuropsychological and pathological assessments. Given the increasing use of machine learning methods to evaluate neuropathology features in the brains of dementia patients, it is important to investigate how the selection of features may be impacted and which features are most important for the classification of dementia. We objectively assessed neuropathology features using machine learning techniques for filtering features in two independent ageing cohorts, the Cognitive Function and Aging Studies (CFAS) and Alzheimer's Disease Neuroimaging Initiative (ADNI). The reliefF and least loss methods were most consistent with their rankings between ADNI and CFAS; however, reliefF was most biassed by feature–feature correlations. Braak stage was consistently the highest ranked feature and its ranking was not correlated with other features, highlighting its unique importance. Using a smaller set of highly ranked features, rather than all features, can achieve a similar or better dementia classification performance in CFAS (60%–70% accuracy with Naïve Bayes). This study showed that specific neuropathology features can be prioritised by feature filtering methods, but they are impacted by feature–feature correlations and their results can vary between cohort studies. By understanding these biases, we can reduce discrepancies in feature ranking and identify a minimal set of features needed for accurate classification of dementia.

## Graphical abstract

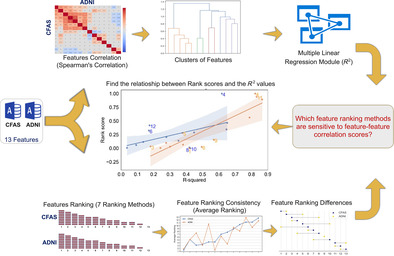

## 1 INTRODUCTION

Dementia poses a significant global challenge, affecting the lives of individuals, their families, and caregivers [1]. The economic burden of dementia was estimated to exceed $818 billion in 2015, and the number of people living with dementia is expected to surpass 75 million by 2030 [2]. Early diagnosis and intervention are crucial in mitigating the negative impact of dementia [3]. However, identifying the determinants of dementia can be difficult because of its complex spectrum of characteristics, encompassing various disorders with distinct pathologies. Alzheimer's disease (AD) is the most common form of dementia, characterised by the presence of amyloid plaques and neurofibrillary tangles in the brain [4].

Feature selection methods play a vital role in biomedical data analysis, helping to identify the most relevant features contributing to a health outcome while eliminating noise, redundancy, and irrelevant factors [5, 6, 7]. Biomedical datasets collected from human biosamples often contain many features, some of which may be irrelevant to the outcome of interest. Analysing all features can lead to overfitting, reduced accuracy, and a less concise understanding of the underlying biological processes [5, 8, 9]. Filter methods measure the relevance of features based on their correlation with the outcome. While commonly used to select features for downstream analysis or machine learning of biomedical datasets, there lacks a systematic comparison of filter methods when used to study complex human disorders, such as dementia.

Previous studies have employed filter methods to identify features related to AD [10, 11, 12]. Gómez‐Ramírez et al. focused on self‐reported data, investigating demographics and other relevant factors associated with the development of dementia from mild cognitive impairment (MCI). Permutation‐based methods were employed as a filter to identify important cognitive decline features. Subsequently, the random forest (RF) algorithm was applied to identify features strongly correlated with cognitive impairment [13]. Thabtah et al. conducted a comprehensive analysis of feature selection methods using continuous features derived from MRI images to detect dementia. The study compared popular methods such as mutual information gain [14], Pearson correlation [15], and symmetric uncertainty [16]. Univariate feature selection and recursive feature elimination techniques were also employed to identify the most informative features correlating with AD using the functional activities questionnaire (FAQ), a common neuropsychological assessment [17, 18]. The authors further investigated the relationships between cognitive and functional features across different levels of dementia progression.

While the relationship between cognitive function and neuropathology features has been extensively explored, less attention has been given to how feature correlation might impact machine learning of dementia. Given the diversity of filter methods and features, it is essential to identify the methods that are less sensitive to similarities or differences between neuropathological features in order to minimise discrepancies in feature rankings. To address these issues, we focused on data from two large dementia studies in the United Kingdom and United States, and hypothesised that there would be associations between feature–feature correlations and the ranking scores computed by filter methods. Several questions arise regarding the ranking of neuropathology features: (1) Which filter methods are less sensitive to feature–feature correlations? (2) Are there differences in feature–feature correlations and rankings between the separate dementia cohorts? (3) How do variations in feature rankings between the cohorts impact dementia prediction models? To investigate these questions, we applied seven filter methods to the two cohort datasets [19, 20, 21] to generate feature rankings and observed how they varied depending on the degree of similarity between features. We are able to identify the best performing feature selection techniques for neuropathology data and assess the level of reproducibility in the associations with dementia found in the two studies.

## 2 MATERIALS AND METHODS

### 2.1 Overview of feature ranking analysis

**FIGURE 1:**
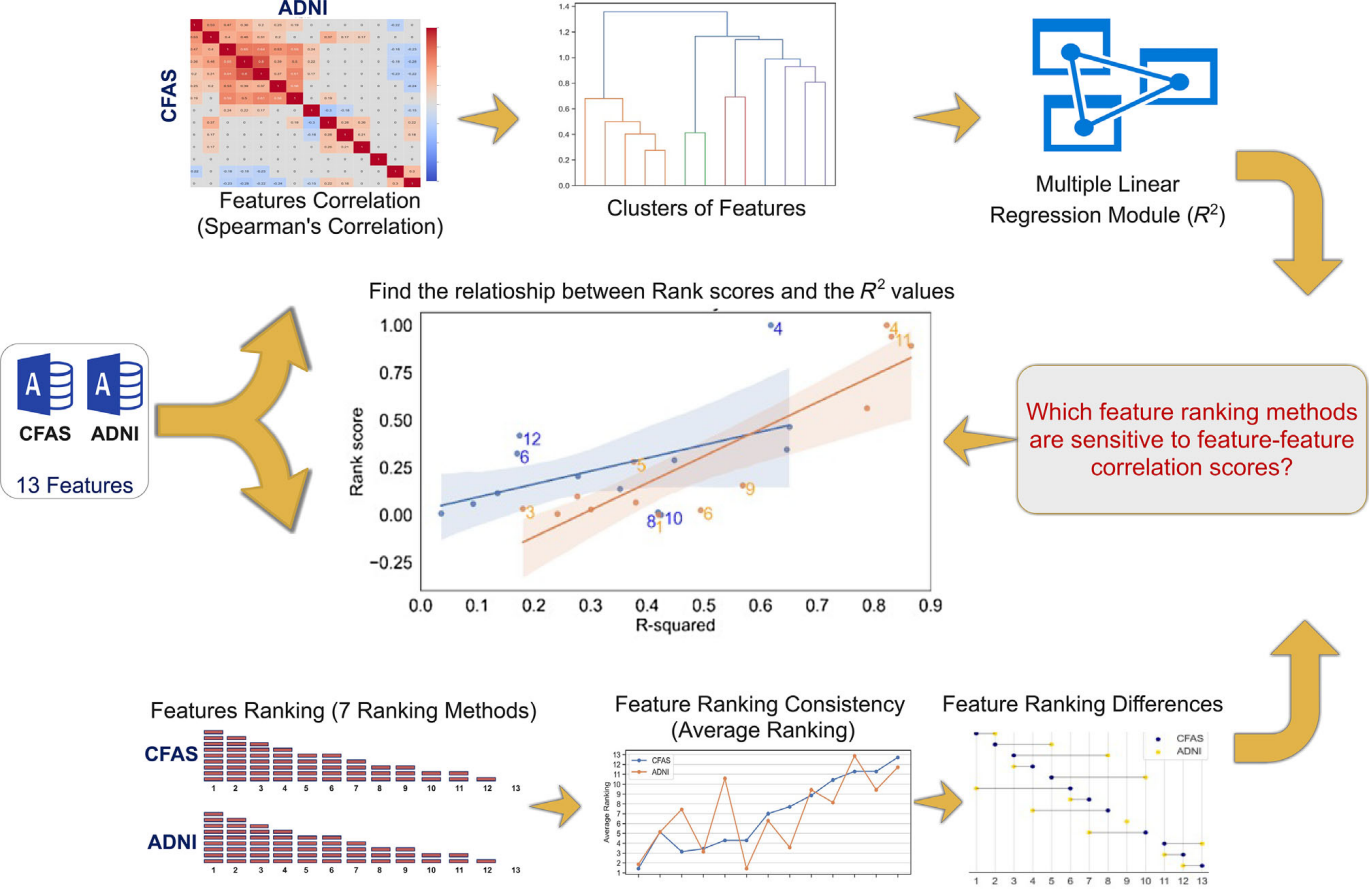
Methodology for dementia classification using CFAS and ADNI datasets. The dementia classification methodology was developed and executed in three key stages: design, implementation, and evaluation. After acquiring neuropathology data, we carried out pre‐processing and determined the correlation between different features. Utilising seven filter methods, we ranked all neuropathological features. Subsequently, we explored the connection between feature–feature correlation and feature ranking across all applied filter methods. Thereafter, classifiers were evaluated using various feature subsets, depending on their interrelations.

We examined the correlation structure of neuropathology and its relationship with dementia (as depicted in Figure 1). Following a comprehensive review and subsequent ethics approval from the management committees, we downloaded the pathological assessments from the Cognitive Function and Ageing Studies (CFAS) [21] and the Alzheimer's Disease Neuroimaging Initiative (ADNI; https://adni.loni.usc.edu/) [22]. After conducting pre‐processing on both datasets, we pinpointed features that were present in both, ensuring their compatibility in terms of features and data types whenever possible. In both datasets, neuropathological features were evaluated and ranked by utilising a range of feature selection techniques centred around various filter methods. We then gauged the ranking disparities between the neuropathological features of CFAS and ADNI, in addition to the consistency between both datasets for each filter method applied. To discover the relationship between a given feature and the remaining ones, we delved into feature–feature correlations using the *R* ^2^ metric, which is based on multiple regression analyses. We took into account both the correlation among the features and their rankings, which was achieved by implementing classification algorithms and noting accuracy, sensitivity, and specificity values. From these insights, we inferred that certain feature subsets can be classified as dementia.

### 2.2 CFAS cohort

This study considered the donated brains of 186 participants, and 13 neuropathological features were assessed (Table S1). These features constituted fundamental neuropathological assessments for each participant, including Braak neurofibrillary tangle (NFT) stage, Thal phase, and cerebral amyloid angiopathy (CAA). Of the total participants, 107 (equivalent to 58%) had been diagnosed with dementia. The participant pool consisted of 72 women and 35 men, with respective median ages of 89 and 88. Among those participants who passed away without a dementia diagnosis (with median ages of 85 for females and 79 for males), the gender distribution was evenly balanced with 37 females and 33 males [23].

### 2.3 ADNI cohort

The data utilised for the creation of this article were acquired from the ADNI database (adni.loni.usc.edu), which was established in 2003 as a collaboration between public and private entities. The neuropathology data version utilised in the ADNI database was NEUROPATH_07_06_21. The ADNI cohort consisted of 1736 individuals, including 85 clinical features across ADNI‐1, ADNI‐GO, and ADNI‐2. For this study, we specifically focused on 80 post‐mortem brains, which exhibited 13 neuropathological features, as detailed in Table S1. These features involved fundamental measures of neuropathology for each subject, such as NFT stage, Thal phase, and CAA. Within the cohort, 77.5% of participants (62 out of 80) were diagnosed with dementia, while 12.5% had MCI, and 10% were cognitively normal (CN). Among the 62 dementia cases, 16 were women and 46 were men, with median ages of 79 and 81.5, respectively. The MCI participants exhibited a gender ratio of 1 female to 9 males (with a median age of 85 for both genders), while those who passed away without a dementia diagnosis had a gender ratio of 5 females to 3 males (with median age of 84 for females and 79 for males). To ensure consistency with the CFAS dataset comparisons, we excluded the 10 participants diagnosed with MCI, leaving us with 70 participants diagnosed with dementia or CN for this study [24, 25, 26].

### 2.4 Feature pre‐processing

In the ADNI dataset, certain features were represented as individual columns, whereas in CFAS, multiple columns were employed to capture related attributes such as ‘infarcts and lacunae’ and ‘diffuse plaques’. Furthermore, CFAS distinguished between infarcts and lacunae separately, whereas ADNI combined them. To address this disparity, our study unified infarcts and lacunae into a single category, treating them as binary indicators of pathology within CFAS. We encountered a similar challenge with the diffuse plaques feature in CFAS, where columns were grouped based on their presence or absence. We refrained from encoding feature measures in both datasets to ensure unbiased feature ranking and analysis. For instance, CFAS utilised a binary representation for cortical atrophy, while ADNI employed an ordinal scale consisting of four categories: no atrophy, mild, moderate, or severe. Upon encoding cortical atrophy as a binary feature, we discovered a 100% correlation between this attribute and certain other features. A similar issue was encountered with ADNI's diffuse plaques feature. Following the pre‐processing stage, we analysed a total of 177 post‐mortems from CFAS and 70 post‐mortems from ADNI, examining 13 neuropathology features for feature ranking.

### 2.5 Ranking neuropathology features

To gain preliminary insight and highlight influential neuropathological features of dementia, we used a variety of feature selection filter methods to measure each feature's relevance. Chi‐square (CHI) [27], gain ratio [28], information gain (IG) [14], reliefF [29, 30], symmetric uncertainty [16], least loss (L2) [31], and variable analysis $V_{a}$ [32, 33] were included in the analysis. Scores varied according to the mathematical criteria and type of filter method used. Because of the different models, there may be discrepancies in the ranking of features based on such scores [33, 34]. Details of the mathematical formulation of the considered filter methods described in the Supplementary Materials (specifically Supplementary Equations 1–10).

The experiment‐related filter‐based feature selection was conducted using Waikato Environment for Knowledge Analysis (WEKA version 3.9.1) [35]. The percentage contribution of each feature was calculated by averaging the total weights assigned by all filter methods to each feature after normalising the weight scores.

### 2.6 Measuring filter methods consistency

Kendall's tau, a measure of correlation between two ranking lists, provides insights into the level of agreement or disagreement between them. Values closer to 1 indicate a stronger agreement, while values closer to −1 indicate a stronger disagreement. A value of tau = 0 suggests no association between the ranking lists. To compare the feature rankings between CFAS and ADNI datasets for each filter method, we utilised the kendalltau() function from the Python3 machine learning package (scipy.stats version 1.7.3). Specifically, we employed this function, available in version v1.9.3, to assess the correlation between the CFAS and ADNI cohorts. The function in SciPy returns *p*‐values based on a two‐tailed test by default. The comparison involved seven filter methods and 13 distinct features.

### 2.7 Imputing missing values

Because of the limitations of the considered cohorts (ADNI = 70 samples, CFAS = 177 samples) and the tendency of machine learning models to encounter errors when encountering NaN values, addressing missing values became necessary. To handle this, we adopted an iterative imputer approach utilising the Scikit‐learn version 0.22.2.post1 [36] library in Python3. This approach allowed us to impute missing values for both numerical and categorical features. For numerical and categorical values, we employed the IterativeImputer from the sklearn.impute package to perform the imputation transformation. To replace missing numeric values, we utilised the RandomForestRegressor from the sklearn.ensemble [37] package as an estimator. The missing values were initially initialised with the mean and underwent a maximum of five iterations. Similarly, for categorical values, we constructed a model employing the RandomForestClassifier from the sklearn.ensemble package. The missing values were initialised with the mean, and the imputation process followed a maximum of five iterations. All the machine learning models and feature selection libraries utilised in this study were developed using Python 3.7.3, ensuring consistency across the analysis.

### 2.8 Measuring feature–feature correlation

In our analysis of CFAS, a total of 177 subjects were included. However, nine subjects had to be excluded from the analysis because of missing values in the class label. Regarding the ADNI dataset, individuals with MCI were excluded, leaving us with a cohort of 70 out of 80 participants who were classified as either CN or diagnosed with dementia. To investigate the relationship between each feature, treated as a dependent variable, and the remaining features, considered as independent variables, we utilised multiple linear regression models. These models were implemented on both the ADNI and CFAS neuropathology cohorts. The coefficients *R* ^2^ obtained from the models (specifically Supplementary Equations 11–13) were used to describe the relationships between the features. To ensure consistency in the analysis, we applied feature normalisation to the numerical features. This was achieved using the minmaxScaler package from scikit‐learn version 0.22.2.post1 [36]. For the linear regression models, we employed the ordinary least squares method with the statsmodels.formula.api package version 0.13.2.

### 2.9 Evaluation of feature ranks against feature–feature correlation

To normalise the scores of the CFAS and ADNI features, we utilised the minmaxScaler package from scikit‐learn version 0.22.2.post1 [36]. This scaling process ensured that the feature scores were within the range of [0, 1]. Consequently, we performed linear regression analyses to examine the relationship between the feature scores and their corresponding *R* ^2^ values for each filter method. For data visualisation and fitting linear regression models, we employed the regplot() function from the Seaborn package version 0.11.0 [38]. The function creates a scatterplot with a linear regression model fit. It employs the Least Squares method to estimate the linear regression coefficients, minimising the sum of the squares of the differences between the observed and predicted values. The function also computes and plots a 95% confidence interval for the regression line, which estimates the uncertainty around the line of best fit. This interval is calculated using bootstrapping with 1000 iterations by default, a resampling method that generates an empirical representation of the sampling distribution and quantifies the uncertainty of the estimate. This allowed us to plot the data and visualise the linear relationship. To calculate the correlation coefficients and corresponding two‐tailed *p*‐values, we utilised the pearsonr() function from the SciPy.stats package version 1.7.3 [39]. This statistical analysis provided valuable insights into the strength and significance of the correlations between the variables.

### 2.10 Dementia classification

In the CFAS dataset, a total of 177 subjects were initially included. However, nine subjects had to be excluded because of missing values in the class label. For the ADNI dataset, individuals with MCI were removed, and the remaining participants with cognitive impairment or dementia were retained. Given the imbalance in the class label of the ADNI dataset, where there were 62 instances of ‘Dementia’ and only 8 of ‘No Dementia’, we utilised the Synthetic Minority Oversampling Techniques for Numerical and Categorical Features (SMOTE‐NC) [40, 41]. This method was applied using the imbalanced‐learn toolbox, version 0.9.1 [42]. This technique involved generating synthetic data instances for the minority class label using the k‐Nearest Neighbours classification algorithm with *k* = 5. After balancing the ADNI dataset, we were left with 124 samples and 13 features. To train and evaluate the classifiers, we utilised scikit‐learn version 0.22.2.post1 in Python3. The evaluation was performed using the ‘leave‐one‐out’ cross‐validation approach, ensuring robustness in the analysis. We also trained classifiers on CFAS samples using ranked features from CFAS and trained classifiers on ADNI samples using ranked features from ADNI. Then we used the trained classifiers to predict dementia status in the other dataset that was held out.

For further assessment of the neuropathological features, we employed various supervised learning techniques, primarily RF [13] and Gaussian Naive Bayes (GNB) [43]. The default parameter settings were used for both RF and GNB. Specifically, RF was configured with 100 estimators (the number of trees in the forest), and the quality of the split was measured using the Gini impurity function. The minimum number of samples required to split a node (min_samples_split) and the minimum number of samples required to be a leaf node (min_samples_leaf) were both set to 1.

### 2.11 Evaluation of classification performance

In this study, we approached the prediction of dementia as a binary classification problem, with the two classes being ‘Dementia’ and ‘No dementia’. To assess the performance of the feature subsets, we employed evaluation metrics such as accuracy, sensitivity, and specificity. These metrics provided valuable insights into the effectiveness of the selected features in predicting dementia. The following evaluation metrics were utilised for performance assessment:

- True positives (TP): Number of dementia cases that were correctly classified.
- False positives (FP): Number of healthy subjects incorrectly classified as dementia cases.
- True negatives (TN): Number of healthy subjects correctly classified.
- False negatives (FN): Number of dementia cases incorrectly classified as healthy subjects.
- Accuracy (%): The proportion of correct classifications among total classifications:
(1) $$\text{Accuracy}=\frac{\mathrm{TP}+\mathrm{TN}}{n},$$
  where *n* is the number of total classifications per test
- Sensitivity (%): The proportion of dementia cases correctly classified
(2) $$\text{Sensitivity}=\frac{\mathrm{TP}}{\mathrm{TP}+\mathrm{FN}}.$$
- Specificity (%): The proportion of healthy subjects correctly classified
(3) $$\text{Specificity}=\frac{\mathrm{TN}}{\mathrm{TN}+\mathrm{FP}}.$$

### 2.12 Code availability and requirements

Links for Python script codes in GitHub for the process and producing all results and figures (https://github.com/mdrajab/CFAS‐and‐ADNI‐Neuropathology.git). The machine was used in this study: macOS Monterey version 12.6.2, MacBook Pro (13‐inch), and Processor: 2.3 GHz Dual‐Core Intel Core i5. Anaconda Navigator 1.9.12 was used to launch Jupyter Notebook version 6.1.4.

### 2.13 Availability of data and materials

Data from the CFAS study is accessible via application to the CFAS (http://www.cfas.ac.uk/cfas-i/data/#cfasi-data-request), under the custodianship of FM and CB. Data from the ADNI study is accessible via application to the ADNI (https://adni.loni.usc.edu/about/), contingent on adherence to the ADNI Data Use Agreement.

## 3 RESULTS

### 3.1 Distribution of neuropathology feature scores across dementia cases

**FIGURE 2:**
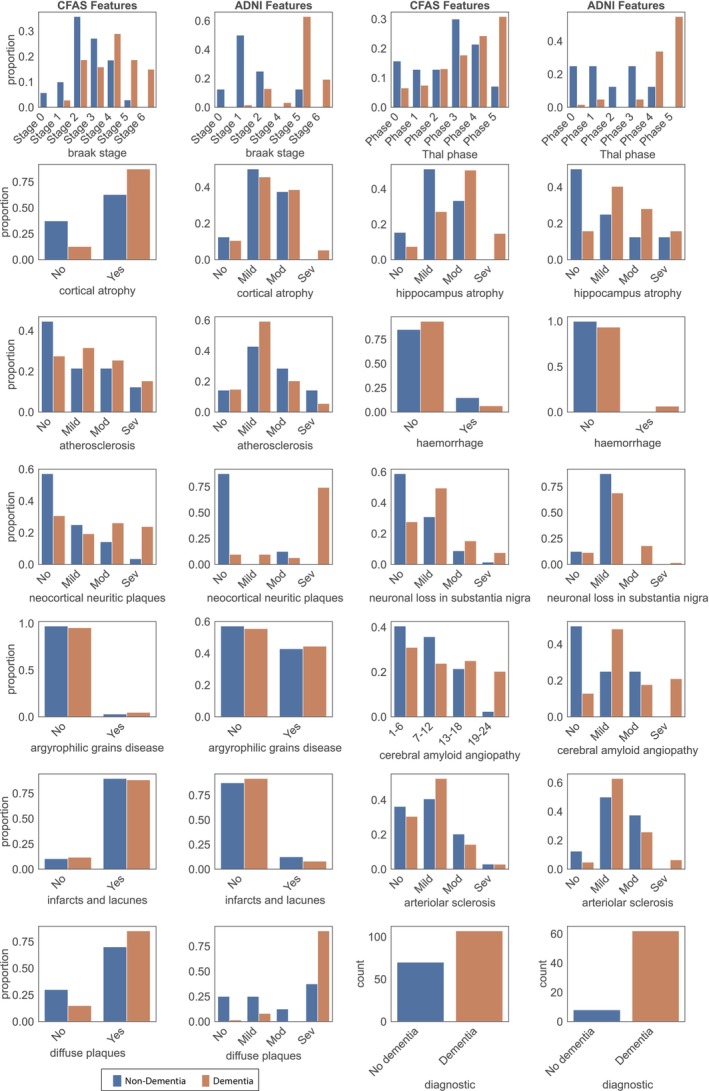
Distribution of neuropathology features in CFAS and ADNI datasets. The distribution of individuals with (orange) and without (blue) dementia was examined in both the CFAS and ADNI neuropathology datasets. The features presented in the table were arranged based on their ranking in the features list, moving from left to right. It is important to note that all features, except for cerebral amyloid angiopathy, were categorical in nature. In CFAS, cerebral amyloid angiopathy was the only feature that had numeric values.

Examining the distribution of neuropathology feature scores among dementia cases was crucial for gaining deeper insights into these features. We conducted an analysis to detect any dissimilarities in the feature distributions between cohorts and to provide plausible explanations for these variations. For this purpose, we plotted the distributions of all neuropathology features for the CFAS and ADNI cohorts, comprising 186 and 70 individuals, respectively (Figure 2). An interesting observation was made regarding the ADNI dataset diffuse plaques feature, which posed a similar challenge as the infarcts and lacunae feature in CFAS. In both cases, we had to group the columns corresponding to diffuse plaques based on their presence or absence. Our findings revealed notable relative differences in the distributions of non‐dementia and dementia cases of certain features in the two cohorts. For instance, cortical atrophy, represented as a binary feature in CFAS and ordinally in ADNI, exhibited a higher proportion of no cortical atrophy in non‐dementia cases. Additionally, other features such as atherosclerosis, neocortical neuritic plaques, neuronal loss in the substantia nigra, argyrophilic grains disease, and diffuse plaques had different shapes of distribution in CFAS and ADNI. These differences in feature distributions may be influenced by a combination of factors, including the varying number of cases in CFAS (*n* = 186) and ADNI (*n* = 70) relative to the small sample size and local differences in diagnosis. These differences are also reflected by the contrasting class distributions within the datasets. Notably, the CFAS dataset displayed a relatively balanced distribution, with 60.5% classified as dementia and 39.5% as non‐dementia, as shown in Table S1. On the other hand, the ADNI dataset exhibited an imbalanced distribution, with 88.6% classified as dementia and 11.4% as non‐dementia. These findings underscore the importance of considering the dataset characteristics, including sample sizes and class distributions when interpreting and comparing the distributions of neuropathology features across cohorts.

### 3.2 Ranking of neuropathology features

**FIGURE 3:**
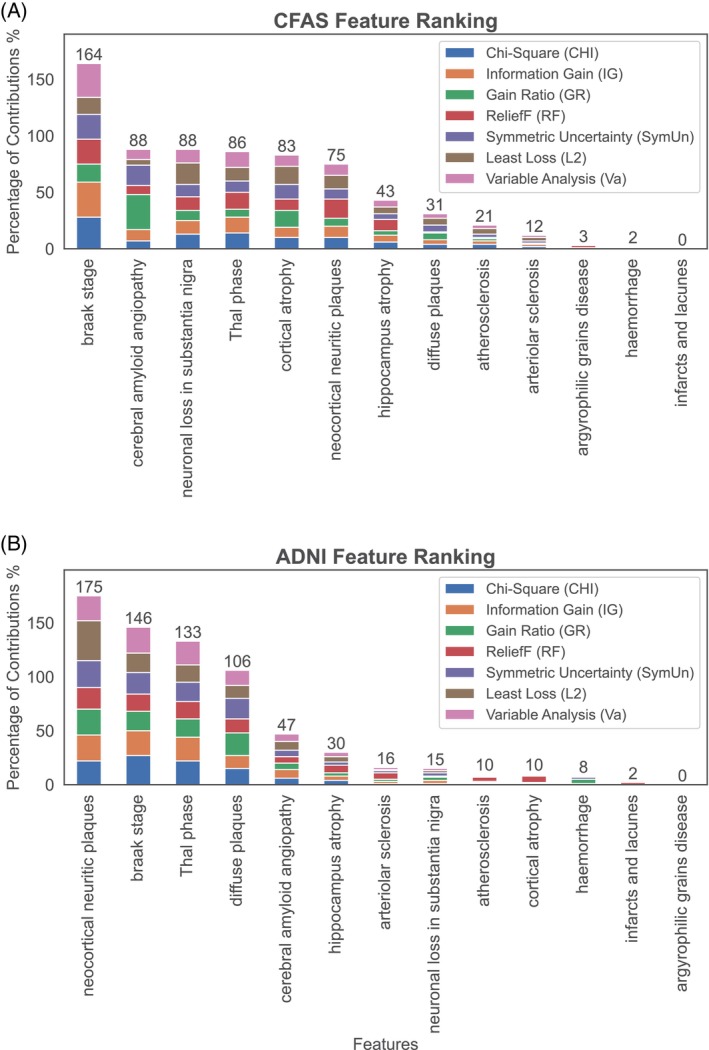
Ranking of neuropathology features in order of association to dementia status as estimated by filter methods in (A) CFAS and (B) ADNI. The cumulative contributions of 13 neuropathology features to dementia status in the CFAS and ADNI datasets are estimated by seven filter methods. The weight scores of each feature were normalised, and the percentage contribution of each feature was calculated by averaging the total weights assigned to it by the filter methods.

To examine the utility of filter methods on dementia‐related features, we conducted a feature selection analysis using two neuropathological datasets, CFAS and ADNI. We aimed to rank the features consistently across both datasets and derive valuable insights for improving dementia diagnosis and treatment. To achieve unbiased and comprehensive results, we employed multiple filter methods to assess the sets of neuropathological features in each dataset. By applying these methods, we calculated feature scores based on the models generated for each filter method (Figure 3). The ranking of features in descending order based on their scores provided a comprehensive and cross‐dataset comparison, aiding the medical profession in better understanding dementia pathology.

The consistent findings across both datasets revealed the importance of certain features in contributing to dementia. The Braak stage emerged as the most influential pathological feature, demonstrating strong correlations, particularly in the CFAS dataset (Figure 3A). In the ADNI dataset, other features such as neocortical neuritic plaques, Thal phase, diffuse plaques, and CAA were also highly correlated with dementia (Figure 3B). Notably, these results were consistent with those obtained from the CFAS dataset, which identified the Braak stage, Thal phase, and CAA as relevant factors associated with dementia. To further investigate the consistency of feature ranking across the filter methods in both CFAS and ADNI datasets, we conducted a detailed analysis. The overall outcomes of our study provided crucial insights into the neuropathological features that play a role in dementia. Furthermore, employing multiple filter methods ensures generalizability and reduces the risk of biassed outcomes, emphasising the importance of considering diverse approaches in feature ranking.

### 3.3 Consistency in the ranking of features between studies

**FIGURE 4:**
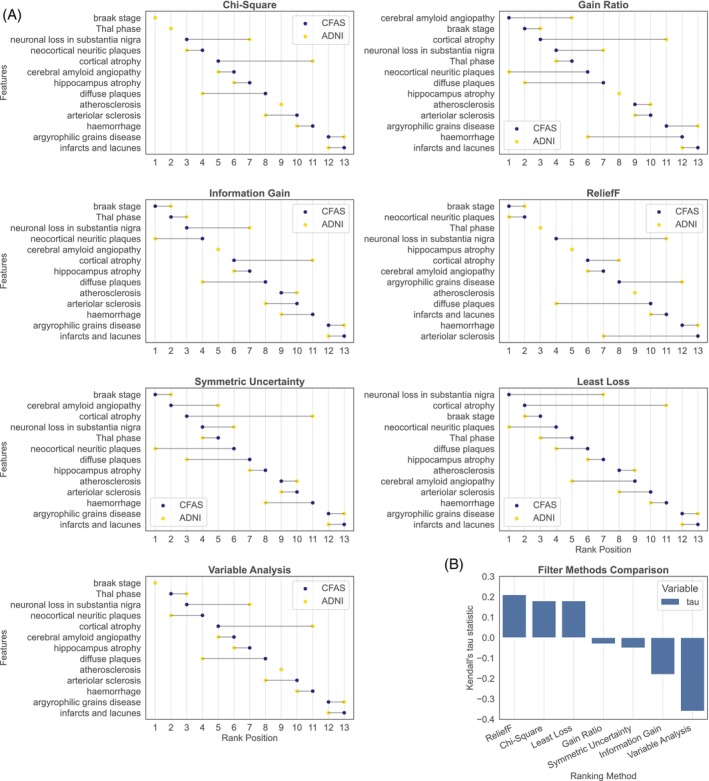
Comparison of feature rankings in CFAS and ADNI. (A) Relative difference in the ranking of each neuropathology feature as estimated by each filter method. (B) Kendall's tau measure of correlation between CFAS and ADNI feature rankings from each filter method.

In this study, pathological feature rankings from the CFAS and ADNI datasets were assessed using various filter methods to identify quantitative discrepancies between these rankings. We compared the positional differences in feature rankings between the two cohort datasets, where the features are arranged based on their rankings in CFAS by each respective filter method (Figure 4A).

Both datasets consistently positioned the top two features (Braak stage and Thal phase) and the ninth feature (atherosclerosis) identically; however, notable differences were found in the rankings of other features. For instance, neuronal loss in the substantia nigra and cortical atrophy occupied the third and fifth positions in CFAS, yet these were ranked four and six positions higher in ADNI. In contrast, diffuse plaques and arteriolar sclerosis, ranked eighth and tenth in CFAS, were higher in ADNI, sitting at fourth and eighth positions.

Evaluation of the filter methods, including information gain, reliefF, symmetric uncertainty, and least loss, revealed considerable variations in feature ranking. Of note, the most prominent discrepancies were identified using the least loss filter method, with the top two features in CFAS and ADNI descending six and nine positions, respectively.

To summarise, there is considerable variability in pathological feature rankings in CFAS and ADNI datasets across the examined filter methods. However, certain features, like the Braak stage, demonstrated consistent patterns irrespective of the filter method employed. The discrepancy in ranking positions might result from differing models used by the filter methods to compute feature‐to‐class correlations. Despite normalising average scores to a unified scale to mitigate deviations, some features displayed diverse rankings.

The study used Figure 4B to compare different filter methods, aiming to showcase the consistency of each method when applied to two datasets, CFAS and ADNI. Kendall's tau measure was used to evaluate the level of consistent feature ranking within each dataset by each filter method. Kendall's tau measures the correlation between two ranking lists, with values near 1 signalling agreement, and values near −1 indicating disagreement. Filter methods were assessed based on their statistical relationships between the ranked features in both datasets.

We show in Figure 4B that the reliefF, chi‐square, and least loss filter methods exhibited positive correlations in feature rankings for both ADNI and CFAS datasets. These results were in line with the earlier feature ranking for these filter methods. For instance, the reliefF, chi‐square, and least loss methods showed similar rankings for features such as Thal phase, Braak stage, neuronal loss in substantia nigra, neocortical neuritic plaque, and cortical atrophy, with minor variations, indicating their reproducibility in feature ranking. Three methods maintained some consistency in feature rankings for the ADNI dataset, including the Braak stage, Thal phase, and neocortical neuritic plaque. Conversely, negative correlations were observed for variable analysis and information gain filter methods with (*r* = −0.36, *p*‐value = 0.10) and (*r* = −0.18, *p*‐value = 0.44), respectively, when applied to ADNI and CFAS datasets. Additionally, the gain ratio and symmetric uncertainty filter methods showed only slight consistency in feature ranking between the ADNI and CFAS datasets, with Kendall correlation coefficients (*r* = −0.03, *p*‐value = 0.95) and (*r* = −0.053, *p*‐value = 0.86), respectively, close to zero. Of all methods, reliefF displayed the highest agreement between CFAS and ADNI feature rankings with Kendall correlation coefficients of *r* = 0.21 and *p*‐value = 0.37, while variable analysis exhibited the most significant disagreement with Kendall correlation coefficients of *r* = −0.36 and *p*‐value = 0.10. Overall, reliefF, chi‐square, and least loss filter methods showed some degree of consistency with Kendall correlation coefficients (*r* = 0.21, *p*‐value = 0.37), (*r* = 0.18, *p*‐value = 0.44), and (*r* = 0.18, *p*‐value = 0.44), respectively, whereas the remaining methods resulted in inconsistent feature ranking between the two datasets.

### 3.4 High correlation between AD pathological features

**FIGURE 5:**
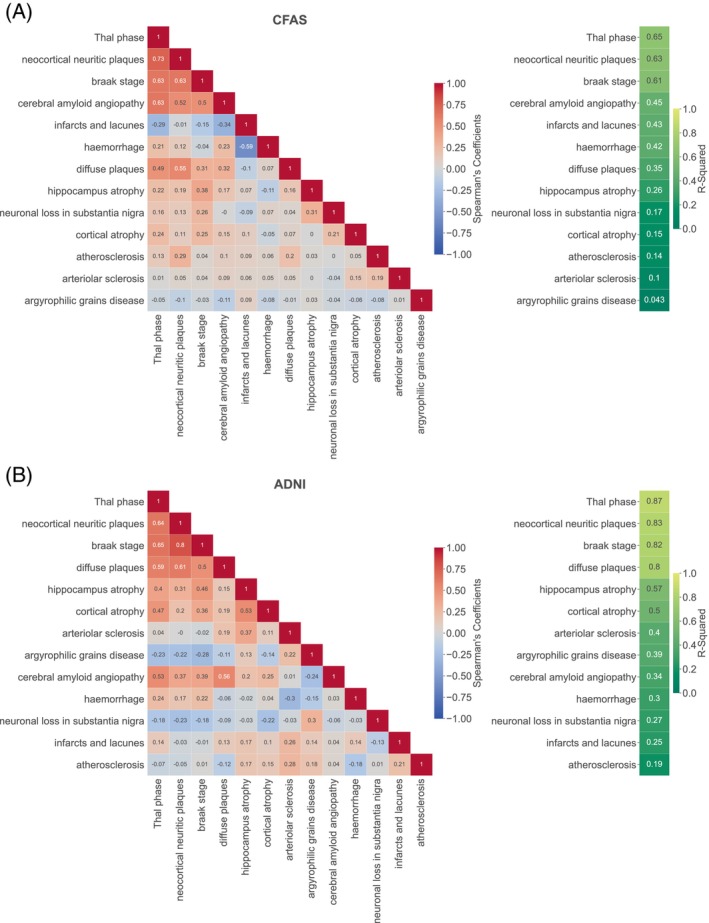
Spearman correlations and *R* ^2^ of pathological features from the ADNI and CFAS datasets. (A) A heat map of Spearman correlation for the CFAS neuropathological dataset. (B) Spearman correlation for the ADNI neuropathological dataset. A correlation coefficient close to 1 (red) indicates a very strong positive correlation between the two variables, while a correlation coefficient closer to −1 (blue) indicates a strong negative correlation. Generally, the lighter the colour, the closer it is to white (zero), and the weaker the correlation. On the right‐hand side of panels, A and B, the *R* ^2^ values range from 0 to 1.

Our study conducted a thorough analysis of the CFAS and ADNI datasets, which included 13 shared features. The analysis involved plotting the feature–feature correlation to identify highly correlated features. For instance, in neocortical neuritic plaques, we observed correlation coefficients of 0.55, 0.52, 0.63, and 0.73 for diffuse plaques, CAA stage, Braak phase, and Thal phase, respectively. Figure 5A shows a positive correlation of Thal phase with diffuse plaques, CAA, and Braak stage with values of 0.49, 0.63, and 0.63, respectively. Additionally, The CAA was positively correlated with diffuse plaques with a value of 0.65, as reflected in Figure 5B. Furthermore, atherosclerosis demonstrated positive correlations with diffuse plaques, CAA, and arteriolar sclerosis of 0.80, 0.64, and 0.61, respectively. CAA also exhibited positive correlations with neuronal loss at substantia nigra and Braak stages of 0.53 and 0.47, respectively. These correlations suggest a possible cluster of features that include the Thal phase, Braak stage, CAA, neocortical neuritic plaques, and diffuse plaques.

Our study aimed to investigate whether the feature–feature correlations substantially influence the feature ranking determined by filter methods and which filter methods were most sensitive to these associations. To achieve this, we excluded the diagnostic class and considered each feature as a dependent variable, and the rest of the features as independent variables. We then utilised multiple regression models to determine the coefficient (*R* ^2^), which measures the similarity of the available feature to the rest of the dataset by identifying how the remaining features can explain the feature's variability. In both CFAS and ADNI, the Thal phase, neocortical neuritic plaque, and Braak stage showed the highest *R* ^2^ scores, indicating their potential significance in these datasets, with scores of 65%, 63%, and 61% for CFAS and 87%, 83%, and 82% for ADNI, respectively. Therefore, to investigate the impact of feature–feature correlations on feature ranking using filter methods, it is necessary to analyse the association between feature correlations and feature ranking scores.

### 3.5 Impact of feature–feature correlations on feature ranking

This study further investigated discrepancies and inconsistencies in feature rankings obtained by filter methods in the two neuropathology datasets. In particular, we considered the impact of feature–feature correlations on feature ranking scores. The aim was to identify filter methods that were less sensitive to potential collinearity between the neuropathological features and observe whether their rankings were more consistent between CFAS and ADNI.

**FIGURE 6:**
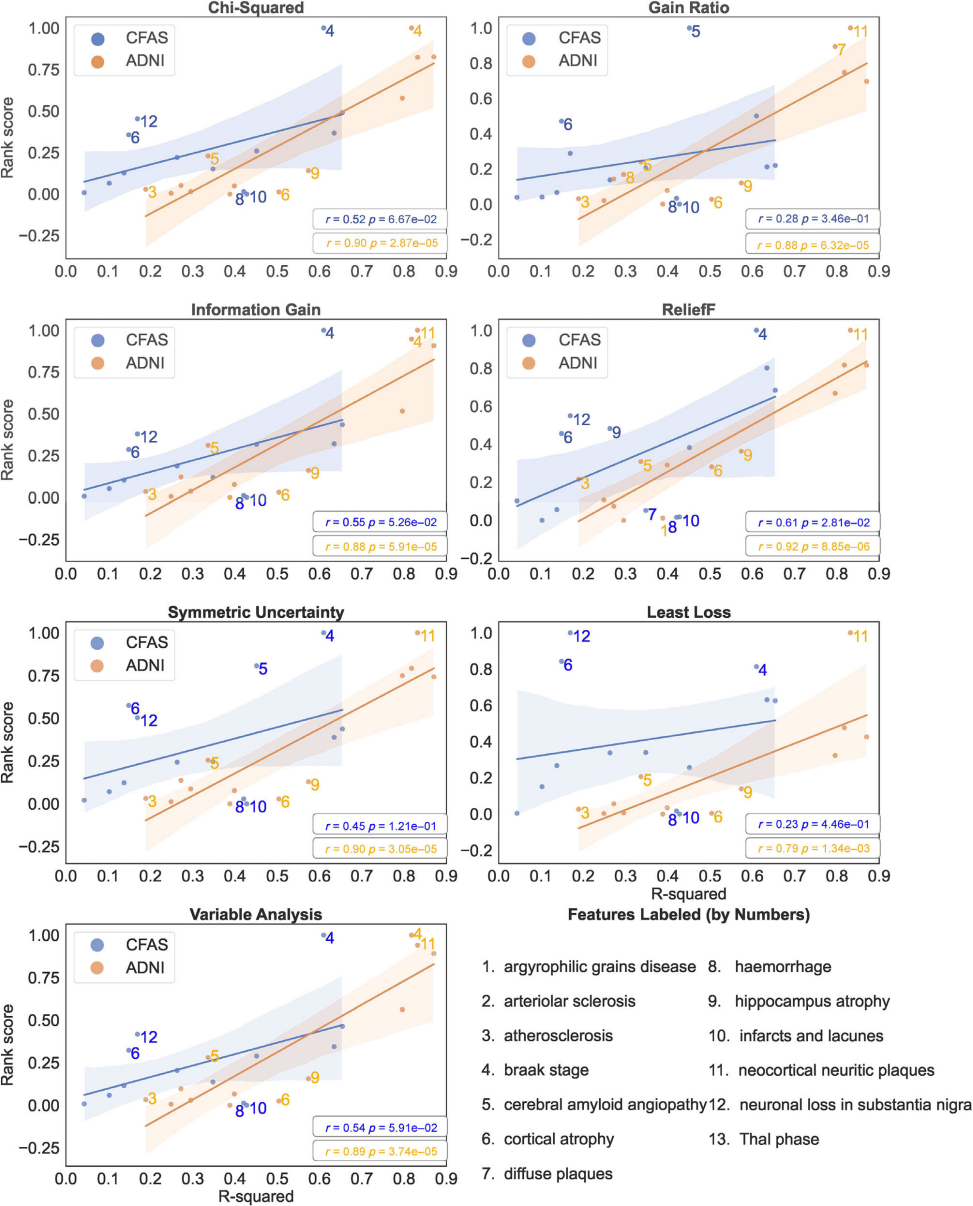
The relationship between feature–feature correlation and feature ranking obtained from filter methods using ADNI‐pathology and CFAS datasets. Best fit linear models with confidence intervals (shading) describe the relationship. Pearson's correlation coefficients (*r*) and *p*‐values (*p*) are reported.

For each dataset, we regressed each feature against all other feature and reported the *R* ^2^ of the fit. We also ranked the features using the different filter methods and applied a Min‐Max normalisation technique on the ranking scores to ensure that all values were on the same scale. For the CFAS dataset, the results indicated a weak positive relationship between feature ranking scores and *R* ^2^ for most filter methods (Figure 6), lowest with Least Loss (*r* = 0.23, *p* = 0.0446) and highest with ReliefF (Pearson *r* = 0.61, *p* = 0.0281). Gain ratio was also demonstrated a weak relationship (CFAS: *r* = 0.28, *p*‐value = 3.46e−01). There was a greater positive correlation between feature ranking scores and *R* ^2^ values in ADNI across all filter methods, with lowest again being Least Loss (*r* = 0.79, *p*‐value = 1.34e−03) and highest with ReliefF (*r* = 0.92, *p*‐value = 8.85e−06).

By illustrating a line of best fit between the rank scores and *R* ^2^, we highlight several features whose ranking are not explained by their feature–feature correlation. In particular, Braak stage achieved a higher rank than expected by its *R* ^2^ when using six of the filter methods in CFAS and three of the methods in ADNI (Figure 6). Cortical atrophy was also higher ranked than expected in all the methods in CFAS, but interestingly in ADNI it was lower than expected. These features may contain less redundant information on dementia status and could be prioritised for neuropathology assessment.

### 3.6 Classification using highly ranked neuropathology features

The study employed classification models, specifically the RF and Naive Bayes classifiers [13], to evaluate the efficacy of selected neuropathological features. We assessed the performance of dementia classification on specific subsets of data from both ADNI and CFAS datasets. The features selected for evaluation in the CFAS and ADNI datasets were presented in Table S2.

**FIGURE 7:**
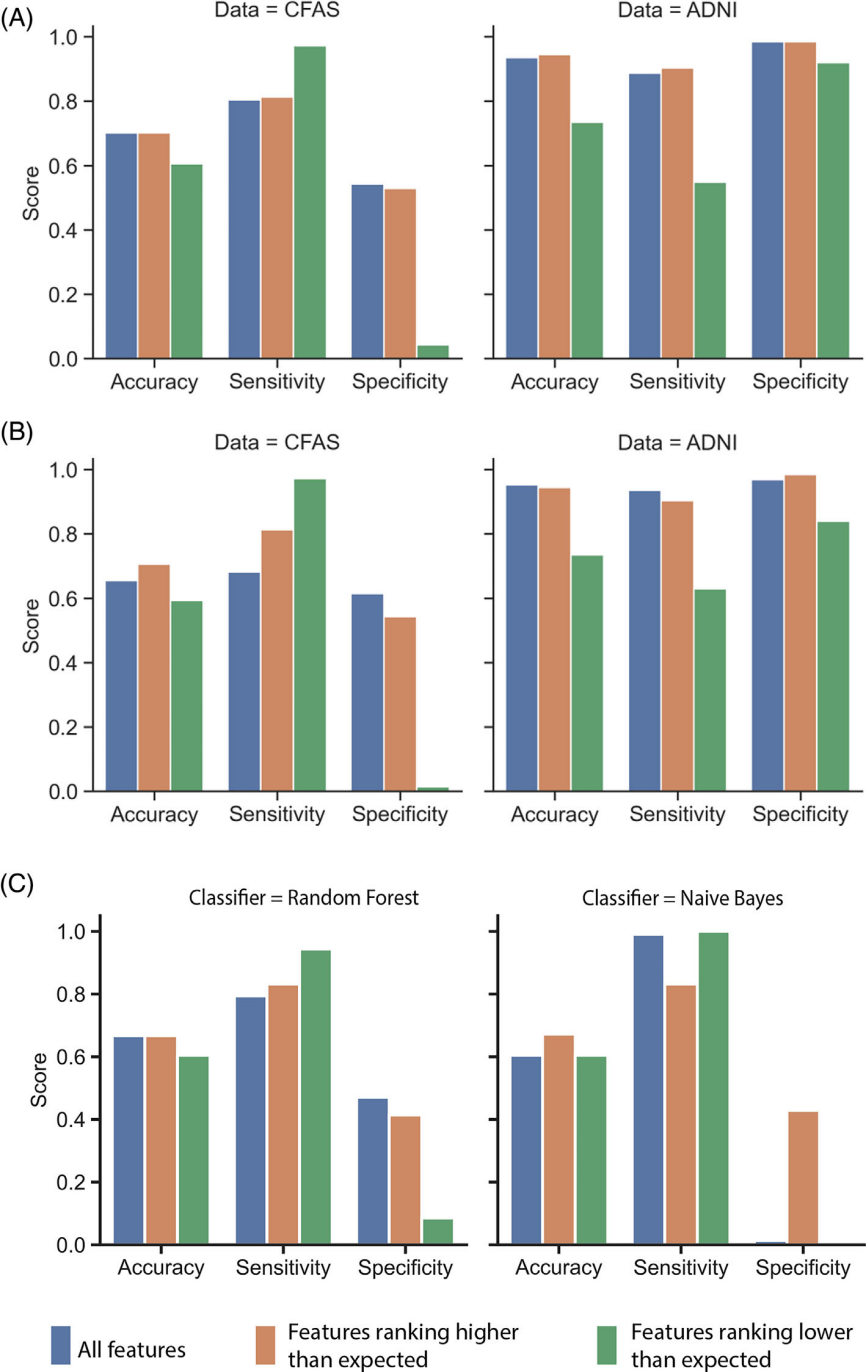
Classification performance using subsets of ranked features. (A) Performance results obtained by the random forest algorithm on the different subsets of features. (B) Performance results obtained by the Naive Bayes algorithm on the different subsets of features. For both classification algorithms, the accuracy, sensitivity, and specificity measures were used for subsets of features for CFAS and ADNI datasets. The feature names for the subsets were shown in Table S2. (C) Performance of the random forest and Naïve Bayes algorithms for classifying individuals in CFAS when trained on the feature rankings and data from ADNI.

Accuracy, sensitivity, and specificity rates of the RF and Naive Bayes classifiers on distinct subsets of neuropathological features in the CFAS and ADNI datasets were investigated and compared. Figure 7A shows that the dementia classifications obtained from the RF algorithm using ADNI in all group subsets were superior to those derived from CFAS, except for the sensitivity rate calculated by the RF algorithm on the CFAS dataset. The same pattern was observed with the Naive Bayes algorithm based on the same group subsets Figure 7B.

The classification algorithms derived from distinct sets of neuropathological features demonstrate higher sensitivity rates. Higher sensitivity rates are desirable to minimise false negatives and ensure that individuals with dementia are accurately identified. Notably, CFAS exhibits remarkable sensitivity rates for features ranking higher than expected (RHE) based on feature–feature correlation. These features are denoted as those falling below the confidence intervals in Figure 6. For instance, the RF classifiers derived from such features in CFAS have a sensitivity rate of 81.3%. A similar trend was observed with Naive Bayes classifiers derived from RHE subset in CFAS with a sensitivity rate of 81.3%.

However, the specificity rate of the CFAS features RHE was low 54.3%. This subset's low specificity implies that the classifiers cannot distinguish patients without dementia from those with the condition. Overall, the classification algorithms and all neuropathological subsets generated low specificity rates, at least for the CFAS dataset.

Conversely, the classification algorithms performed exceptionally well for distinct subsets of the neuropathological features in ADNI. According to the ADNI results, a RF algorithm produced the best classifier for features RHE, with a 94.40% accuracy, 90.0% sensitivity, and 98.4% specificity, demonstrating high predictive power. While in CFAS, Naive Bayes performed best for a subset of ranking higher than expected at 70.6% accuracy, 81.3% sensitivity, and 54.3% specificity. When we trained the classifiers on ADNI, the classification performance for RF dipped only slightly to 67.0% accuracy and for Naïve Bayes to 67.2% accuracy using the highly ranked features (Figure 7C).

The feature selection analysis results align with the classification algorithms' findings. Based on these results, clinicians can leverage informative neuropathological features during the clinical assessment of dementia, including features RHE by feature–feature correlation from ADNI. Furthermore, although the sensitivity results obtained from the distinct feature subsets of CFAS by the classification algorithm were remarkable, the ADNI feature subset results were more convincing. This was because the performance measures results were balanced, making ADNI more suitable for dementia analysis, at least when neuropathological features were considered.

## DISCUSSIONS AND CONCLUSIONS

According to the initial analysis presented in the distribution of neuropathology feature scores across dementia cases, several findings were observed, some of which have been previously published. Both the CFAS and ADNI studies showed that the percentage of individuals with dementia increased as the Braak stage increased, with a peak at stage IV for CFAS and stage V for ADNI [20, 44]. Similarly, both studies observed an increase in the Thal phase [20, 44]. The CFAS data showed higher CAA across individuals, while ADNI revealed a higher rate of dementia was associated with a higher number of brain areas with CAA and its severity. Additionally, both studies observed brain atrophy in dementia patients.

The study demonstrated consistent rankings of neuropathological features across different filter methods, with Kendall's tau revealing correlated rank orders between ADNI and CFAS datasets for methods generating the same rankings. Notably, reliefF, chi‐square, and least loss methods exhibited similar rankings for specific features in both datasets, suggesting their reproducibility. However, some methods, such as gain ratios and symmetric uncertainty, resulted in divergent rankings between ADNI and CFAS. The observed variability in rankings may be attributed to differing models used by these methods for feature‐to‐class correlations. Despite deviations, certain features, like the Braak stage, maintained consistent patterns across filter methods. Figure 4B highlighted consistency in rankings from reliefF, chi‐square, and least loss methods in both datasets, underscoring their reliability. ReliefF demonstrated the highest agreement between CFAS and ADNI feature rankings, but its rankings were mostly explained by feature–feature correlation. This method calculates feature weights based on differences in feature values between instances and their nearest neighbours. When two or more features are highly correlated, the differences in their values may not provide distinct and meaningful information. In contrast, using least loss for feature ranking may be less impacted by feature–feature correlation because it selects features that contribute the least to a classifier model's loss or error, hence, focusing more on the marginal information gained by the feature. Recommendations from filter methods seemed context‐dependent, so future analyses should explore sensitivity analyses, techniques to normalise different feature distributions, and ensemble approaches to assess the suitability of combining different methods for specific cohorts in order to enhance the robustness and generalizability of the findings.

We further assessed the impact of selecting subsets of ranked features on the classification of dementia. Classification algorithms developed from distinct sets of neuropathological features had a high cross‐validation accuracy of up to 94.4%, 90.3% sensitivity, and 98.4% specificity using the RF classifier in ADNI for ranked features impacted by feature–feature correlation. While in CFAS, the Naive Bayes classifier achieved the highest cross‐validation performance in classifying dementia status with 70.6% accuracy, 81.3% sensitivity, and 54.3% specificity for the subset of highly ranked features. The performance on CFAS classification is similar (67.2% accuracy) when Naive Bayes was trained on ADNI. This classification performance is consistent with the previous classification models using neuropathology features in CFAS [20], and using imaging features from ADNI in deep neural networks [45, 46].

The study's limitation stems from differing dementia prevalence in the cohorts, posing a potential confounder for interpreting neuropathological feature correlations. The observed associations may partly reflect the influence of varying dementia rates rather than intrinsic feature relationships. Generalizability is constrained by cohort‐specific characteristics, cautioning against broad extrapolations, especially to populations with distinct dementia neuropathological feature distributions. To mitigate the impact of imbalanced class distribution, we employed SMOTE‐NC during the resampling process. Future research should consider stratified analyses based on dementia status, explore methods to further mitigate confounding, and prioritise larger, balanced samples for robust conclusions in dementia‐related neuropathological investigations. Addressing missing data became imperative in our study because of the limitations in cohort sizes (ADNI = 70 samples, CFAS = 177 samples) and the potential challenges for machine learning models in handling NaN values. To tackle this issue, we implemented an iterative imputation approach using the Scikit‐learn library. This method facilitated the imputation of missing values for both numerical and categorical features. Our meticulous imputation strategy was crucial not only caused by the inherent limitations in our cohorts but also in the context of the preceding discussion on the impact of missing value imputation on feature–feature correlations, especially in the domain of dementia neuropathology data.

In conclusion, this research demonstrated the association between feature–feature correlation and the feature ranking scores obtained by filter methods in medical applications such as dementia diagnosis. The study found that the ReliefF filter method is less sensitive to feature–feature correlations and that these correlations substantially impact the ranking of features and the performance of diagnosis models developed from the two dementia cohorts. The findings of this study indicate that filter methods for selecting neuropathology features associated with dementia are impacted by the feature–feature correlation and may differ between cohort studies. It is important to note that these results are based on the analysis of just two datasets, and further study may be required for broader applicability. By investigating the potential bias in filter methods, it is possible to minimise discrepancies in feature rankings and determine a reliable set of important features for the purpose of classification algorithms.

## AUTHOR CONTRIBUTIONS

*Data analysis*: MR, DW. *Writing of first draft*: MR, DW. *Common Features between CFAS and ADNI*: MR, TT, DW. *Data oversight and analysis results interpretation*: MR, DW. *Contribution to interpretation and to the final manuscript*: MR, DW. All authors read and approved the final manuscript.

## FUNDING INFORMATION

This work was supported by the Medical Research Council (MRC/G9901400, U.1052.00.0013, G0900582). SBW is also supported by the Alzheimer's Society (AS‐PG‐17‐007 and AS‐PG‐14‐015). Work in the individual CFAS centres is supported by the NIHR Sheffield Biomedical Research Centre—awarded to Newcastle‐upon‐Tyne Hospitals Foundation Trust; Cambridge Brain Bank supported by the NIHR Cambridge Biomedical Research Centre; Nottingham University Hospitals NHS Trust; University of Sheffield, Sheffield Teaching Hospitals NHS Foundation Trust and NIHR Sheffield Biomedical Research Centre; The Thomas Willis Oxford Brain Collection, supported by the Oxford Biomedical Research Centre; The Walton Centre NHS Foundation Trust, Liverpool. DW is supported by the Academy of Medical Sciences Professorship (APR7_1002) and the Engineering and Physical Sciences Research Council (EP/V029045/1). MR is supported by the Saudi Arabia Ministry of Education, PhD Scholarship. We would like to acknowledge the essential contribution of the liaison officers, the general practitioners, their staff, and nursing and residential home staff. We are grateful to our respondents and their families for their generous gift to medical research, which has made this study possible.

## CONFLICT OF INTEREST STATEMENT

The authors declare no conflicts of interest.

## CONSENT STATEMENT

For the CFAS dataset, fully written informed consents were obtained from all participants or their authorised representatives, and the study was conducted in accordance with the ethical standards of the Declaration of Helsinki. The study was undertaken with ethical approval from a UK Multicentre Research Ethics Committee (10/H0304/61).

## Supporting information


**Data S1.** Supporting information.

**Table S1.** Neuropathology features from the CFAS and ADNI cohorts considered for feature selection.

**Table S2.** Selected sets of features from the CFAS and ADNI Datasets based on feature ranking and feature–feature correlations.

## Data Availability

Data from the CFAS study is accessible via application to the CFAS (http://www.cfas.ac.uk/cfas-i/data/#cfasi-data-request), under the custodianship of FM and CB. Data from the ADNI study is accessible via application to the ADNI (https://adni.loni.usc.edu/about/), contingent on adherence to the ADNI Data Use Agreement.
