# Supplementary material for: Ranking and filtering of neuropathology features in the machine learning evaluation of dementia studies
Source: Brain Pathol. 2024 Feb 19;34(4):e13247. doi: 10.1111/bpa.13247 (PMC11189772; doi:10.1111/bpa.13247)
Supplement: Supplementary file 1 — Data S1. Supporting information. [file BPA-34-e13247-s002.docx]

Supplementary Materials

## **Ranking Neuropathology Features**

Details of the mathematical formulation of the considered filter methods follow:

Chi-square (CHI) utilises the difference between observed and expected frequencies of the instances as shown in Equation (1). The formula is given by:

$X^{2}=\sum_{i=1}^{n} \frac{\left( {Observed}_{i} -{Expected}_{i} \right)^{2}}{{Expected}_{i}}$ , (1)

where $X^{2}$ is the chi-square statistic. ${Observed}_{i}$ is the observed frequency for a specific feature in category $i.{Expected}_{i}$ is the expected frequencies for a specific feature in category $i$ under the assumption of independence.

Information gain (IG) employs Shannon entropy to measure the correlation between a feature and dementia status (Equations 2 and 3). This is a measure of how well a feature separates a dataset into subsets of more homogenous classes. It's often calculated as the difference between the entropy of the original dataset and the weighted sum of entropies of the subsets formed by the split. The formula for IG is given by:

$IG (S, F) = Entropy (S) -\Sigma_{v\in Values(F)}\frac{|S_{v} |}{| S |} \cdot Entropy (S_{v})$, (2)

where $IG (S, F)$ donates as Information Gain for dataset $S$ and feature $F$. $Entropy (S)$ is the entropy of the dataset $S$ before the split (Equation 3). It measures the uncertainty or impurity in the dataset. $Values(F)$ is the set of all possible values that feature $F$ can take. $|S_{v} |$ is the size of the subset of $S$ for which feature $A$ has the value $v$. $| S |$ is the total size of the dataset $S$. $Entropy (S_{v})$ is the entropy of the subset $S_{v}$ after the split based on feature $F$ (Equation 3).

The Shannon entropy formula is a measure of uncertainty or information content in a set of data. The interpretation of entropy is that it represents the average amount of information needed to specify the class of an instance drawn randomly from the dataset. A higher entropy indicates higher uncertainty or disorder in the dataset. The formula for Shannon entropy is given by:

$Entropy(S)= -\Sigma_{i=1}^{c}P_{i} \cdot{log}_{2}{(P}_{i})$, (3)

where $Entropy(S)$ is the Shannon entropy of the dataset $S$. $c$ is the number of classes in the dataset *S*. $P_{i}$is the probability of occurrence of class i in the dataset *S*. ${log}_{2}{(P}_{i})$ is the logarithm base 2 of the probability $P_{i}$.

Gain ratio is a normalised form of IG which is estimated by dividing the IG with the entropy of the feature with respect to the class (Equations 4 and 5). The Gain Ratio helps in selecting features that provide a good balance between information gain and the potential complexity introduced by creating multiple branches. Features with a higher Gain Ratio are considered more informative for splitting the dataset.

$Gain Ratio=\frac{IG}{Entropy(S,F)}$, (4)

where IG denotes the information gain (Equation 2), and $Entropy(S,F)$ is the shannon entropy of the subset $S$ with respect the feature $F$ (Equation 5). It measures the uncertainty or disorder in the subset, considering the values of the specific feature.

$Entropy(S,F)= -\Sigma_{i}\left( \frac{S_{i}}{S} {\cdot log}_{2} \left( \frac{S_{i}}{S} \right) \right)$, (5)

where $Entropy(S,F)$ is the Shannon entropy of the subset $S$ with respect to the feature $F$. The summation symbol $\Sigma_{i}$indicates the sum over all possible values of the feature $F$. $S_{i}$ is the size of the subset $S$ where the feature $F$ has a specific value $i$. $S$ the total size of the subset $S$. $\frac{S_{i}}{S}$ is the probability of an instance in the subset having the specific value $i$ for the feature $F$. It's essentially the ratio of instances with that value to the total number of instances in the subset. ${log}_{2} \left( \frac{S_{i}}{S} \right)$ This term measures the amount of information associated with the specific value $i$ of the feature $F$. The logarithm is used to convert the probability into information content measured in bits. The overall entropy for the subset $S$ with respect to the feature $F$ is then calculated by summing these entropies over all possible values of the feature. The negative sign ensures that the entropy is a positive value and follows the convention of entropy being a measure of uncertainty or disorder.

Symmetric uncertainty (SU) deals with the bias of IG that occurs due to a large number of distinct values for the feature and presents a normalised score (Equation 6). Symmetric Uncertainty provides a normalised measure of mutual information between two features, taking into account the information gain and the entropies of each feature. This metric is useful in feature selection and understanding the relationships between different features in a dataset.

$SU(A,B) = \frac{2 \times IG(A|B)}{Entropy\left( A \right) + Entropy(B)}$, (6)

where $SU(A,B)$ measures the mutual information or shared information between variables $A$ and $B$. $IG(A|B)$ is the Information Gain of variable $A$ given the value of $B$ after knowing the class. It measures how well variable $B$ helps in reducing uncertainty about variable $A$. $Entropy(A)$ is the entropy of variable $A$. It quantifies the amount of uncertainty or disorder in variable $A$. $Entropy(B)$ is the entropy of variable $B$. It quantifies the amount of uncertainty or disorder in variable $B$.

ReliefF calculates the scores of each available feature with the class using the differences between the neighbouring data instances and the target instances (Equation 7). The basic idea behind ReliefF algorithms is to estimate the importance of features based on their ability to distinguish between instances of the same and different classes. The ReliefF algorithm considers both nearest hits and nearest misses to assign feature weights.

$W\left[ F \right]=W\left[ F \right]-\frac{\Sigma_{i=1}^{k} \delta\left( F, R_{i}, H \right)}{k}+\frac{\Sigma_{i=1}^{k} \delta\left( F, R_{i}, M \right)}{k}$ , (7)

where $W\left[ F \right]$ is the weight associated with feature F. $k$ is a user-defined parameter representing the number of nearest neighbours to consider. $R_{i}$ is a randomly chosen instance. $\delta\left( F,R_{i}, H \right)$represents the difference or distance between feature $F$ in the instance $R_{i}$ and the nearest instance of the same class (hit). $\delta\left( F,R_{i}, M \right)$ represents the difference or distance between feature $F$ in the instance $R_{i}$ and the nearest instance of a different class (miss). The algorithm iteratively updates the weights for each feature by considering the differences for both hits and misses and normalises the weights at the end.

The Least Loss (L^2^) method is computed per feature using the squared difference between the joint probabilities and the product of the marginal probabilities of two variables Y and X. This formula quantifies the dissimilarity or "loss" between the observed and expected joint probabilities of Y and X. The method employs a vector of scores derived from both CHI (Chi-square) and IG (Information Gain) results for Variable Analysis V_a_ (see Equation 9 and 10). These scores are then normalised, and the vector magnitude is computed using Equations 9 and 10. This aims to capture the interplay and significance of features within the dataset. Equations 8, 9, and 10 collectively form a comprehensive framework for analysing and selecting features based on the L^2^ method.

$L^{2}\left( Y,X \right)= \Sigma_{i,j}{[\hat{p}\left( y_{i} ,x_{j} \right)-\hat{p}{(y}_{i})\hat{p}(x_{j})]}^{2}$, (8)

where $X$ is the independent feature class, $Y$ is the class label. $L^{2}\left( Y,X \right)$ is the least loss between the $Y$ and $X$ based on the calculated squared differences between joint and marginal probabilities. The assumption symbol $\Sigma_{i,j}$is performed over all possible values of the variables $Y$ and $X$. $\hat{p}\left( y_{i} ,x_{j} \right)$ represents the joint probability of the occurrence of values $y_{i}$ and $x_{j}$. $\hat{p}{(y}_{i})$ represents the marginal probability of the occurrence of $Y_{i}$. $\hat{p}(x_{j})$ represents the marginal probability of the occurrence of $x_{j}$.

The score vector comprises information from both the IG and CHI scores. It's important to remember that the magnitude of a vector is determined by taking the square root of the sum of squares of its coordinates. Consequently, the magnitude of the score vector serves as a scalar metric for the vector. The score vector's magnitude facilitates the comparison of features, assigning higher rankings to those with greater values.

$V_{a}=\left( \frac{{IG}_{a}}{{CHI}_{a}} \right)$, (9)

where $\frac{{IG}_{a}}{{CHI}_{a}}$ represents the ratio of the Information Gain ${IG}_{x}$ to the Chi-Square scores along the $a$th feature. This ratio is used to quantify the relationship or proportionality between these two measures in a specific context. This kind of ratio can be useful in certain contexts, particularly in feature selection or variable analysis, to understand the relative importance or relevance of Information Gain and Chi-Square measures for a given variable along the $a$th feature.

$\left| V_{a} \right|=\sqrt{{({IG}_{a})}^{2}+{({CHI}_{a})}^{2}}$, (10)

where this represents the square root of the sum of the square of its CHI and IG results of $a$th feature. The square root of the sum of squares yields the magnitude (absolute value) of the vector $V_{a}$. Combining and standardising CHI and IG scores produces a new feature rank, improving the identification of crucial features during preprocessing. This directly influences phishing detection, and the removal of less significant features offers benefits such as reduced data dimensionality and a streamlined search space for efficient training without sacrificing classifier performance.

## **Measuring Feature-feature Correlation**

The determination of coefficient (R^2^) represents the similarity of the dependent feature with the independent features by showing to which level the remaining features can explain the variability of the feature at hand.

$R^{2} = 1 -\frac{SSR}{SST}$, (11)

where $SSR$ is the sum of squares residuals (Equation 12) and $SST$ is the total sum of squares (Equation 13). $\frac{SSR}{SST}$ expresses the proportion of the total variance in the dependent variable that is not explained by the model

$SSR =\sum_{i=1}^{n} (y_{i} - f(x_{i}))^{2}$, (12)

where $n$ is the number of observations, $y_{i}$ is the i^th^ value of the feature to be predicted, and ${f(x}_{i})$ is the predicted value of $y_{i}$. In regression analysis, this is to minimise the sum of squares of residuals. A smaller SSR indicates a better fit of the model to the observed data, meaning that the predicted values are closer to the actual observed values.

$SST =\sum_{i=1}^{n} (y_{i} - \underline{y})^{2}$, (13)

where $n$ is the number of observations, $y_{i}$ is the the value in a sample, and $\underline{y}$ is the mean value of a sample. The total sum of squares measures the total variability in the dependent variable. It serves as a benchmark against which the explained variability by a regression model (captured by the sum of squares of residuals, $SSR$) can be compared.
