# Supplementary material for: Ranking and filtering of neuropathology features in the machine learning evaluation of dementia studies
Source: Brain Pathol. 2024 Feb 19;34(4):e13247. doi: 10.1111/bpa.13247 (PMC11189772; doi:10.1111/bpa.13247)
Supplement: Supplementary file 2 — Table S1. Neuropathology features from the CFAS and ADNI cohorts considered for feature selection. [file BPA-34-e13247-s003.docx]

**Table S1**: Neuropathology features from the CFAS and ADNI cohorts considered for feature selection.

| **No** | **Feature** | **Feature Description** | **CFAS** | | | | **ADNI** | | | |
| --- | --- | --- | --- | --- | --- | --- | --- | --- | --- | --- |
|  |  |  | **Type** | **Dementia**  **(n=107)** | **No Dementia (n=70)** | **Missing (n=9)** | **Type** | **Dementia**  **(n=62)** | **No Dementia (n=8)** | **Missing (n=0)** |
| **1** | Braak stage | Braak neurofibrillary tangle (NFT) stage[[27,28]](https://paperpile.com/c/JTKcnK/6KgeB+oGuSQ). | Nominal | 107 | 70 | 0 | Nominal | 62 | 8 | 0 |
| **2** | Thal phase | Detects immunopositive amyloids in cortical and subcortical areas[[29,30]](https://paperpile.com/c/JTKcnK/MZ3s4+wui5K). | Nominal | 107 | 70 | 0 | Nominal | 62 | 8 | 0 |
| **3** | Cortical atrophy | A condition in which the brain’s cortex—the outer layer of the cerebrum––thins and shrinks in size. | Binary | 103 | 67 | 7(4.0%) | Nominal | 57 | 8 | 5(7.1%) |
| **4** | Hippocampus atrophy | Characterised by a decrease in the size of the hippocampus, the area of the brain responsible for the formation and recall of memories. | Nominal | 81 | 39 | 57(32.2%) | Nominal | 57 | 8 | 5(7.1%) |
| **5** | Atherosclerosis | A condition that is characterised by the hardening and narrowing of arteries due to a buildup of fatty deposits known as plaque. | Nominal | 98 | 65 | 14(7.9%) | Nominal | 54 | 7 | 9(12.9%) |
| **6** | haemorrhage | A medical condition in which there is a loss of blood. | Binary | 47 | 27 | 103(58.2%) | Binary | 62 | 8 | 0 |
| **7** | Neocortical neuritic plaques | Accumulation of amyloid beta peptides in the brain in the form of neuritic plaques, comprising dense deposits of amyloid beta protein. | Nominal | 88 | 56 | 33(18.6%) | Nominal | 62 | 8 | 0 |
| **8** | Neuronal loss in substantia nigra | Characterised by the death of neurons in the substantia nigra, a part of the brain associated with movement and coordination. | Nominal | 105 | 68 | 4(2.3%) | Nominal | 61 | 8 | 1(1.3%) |
| **9** | Argyrophilic grains disease | A type of tauopathy which is a class of neurodegenerative diseases caused by an accumulation of the tau protein in the brain. | Binary | 107 | 69 | 1(0.6%) | Binary | 27 | 7 | 36(51.4%) |
| **10** | Cerebral amyloid angiopathy (CAA) | A form of cerebrovascular disease in which amyloid protein deposits accumulate in the walls of small blood vessels in the brain. | Numeric | 84 | 42 | 51(28.8%) | Nominal | 62 | 8 | 0 |
| **11** | Infarcts and lacunes | Types of brain lesions that are commonly associated with a stroke. Infarcts are areas of tissue death caused by a lack of oxygen due to a blockage of the brain's blood vessels. Lacunes are small cavities that develop when parts of the brain become damaged or die due to a lack of oxygen or other factors. Often caused by small strokes or other vascular changes. | Binary | 51 | 29 | 97(54.8%) | Binary | 62 | 8 | 0 |
| **12** | Arteriolar sclerosis | A condition in which the walls of the arterioles become stiff and thickened due to deposits of fatty material. | Nominal | 105 | 69 | 3(1.7%) | Nominal | 62 | 8 | 0 |
| **13** | Diffuse plaques | Caused by deposits of amyloid-beta proteins accumulating in the brain. These proteins form clumps that disrupt normal cell functioning, leading to inflammation and damage. | Binary | 107 | 70 | 0 | Nominal | 62 | 8 | 0 |
| **14** | Diagnostic | Class label (dementia or no dementia) status of a patient | Binary | 107 | 69 | 0 | Binary | 62 | 8 | 0 |
