# Supplementary material for: Ranking and filtering of neuropathology features in the machine learning evaluation of dementia studies
Source: Brain Pathol. 2024 Feb 19;34(4):e13247. doi: 10.1111/bpa.13247 (PMC11189772; doi:10.1111/bpa.13247)
Supplement: Supplementary file 3 — Table S2. Selected sets of features from the CFAS and ADNI Datasets based on feature ranking and feature–feature correlations. [file BPA-34-e13247-s001.docx]

**Table S2:** Selected sets of features from the CFAS and ADNI Datasets based on feature ranking and feature-feature correlations.

| **Description** | **CFAS** | **ADNI** |
| --- | --- | --- |
| All features | All 13 features | All 13 features |
| Features ranking higher than expected (RHE)⁺ | 1. Braak stage 2. Cortical atrophy 3. Neuronal loss in substantia nigra | 1. Atherosclerosis 2. Braak stage 3. CAA 4. Neocortical neuritic plaques |
| Features ranking lower than expected (RLE)⁻ | 1. Haemorrhage 2. Infarcts & Lacunes | 1. Hippocampus 2. Cortical atrophy 3. Argyrophilic grain disease |

⁺ Features ranking higher than expected: Features that rank higher than expected, based on feature-feature correlation, are denoted as the features that fall below the confidence intervals in Figure 6.
⁻ Features ranking lower than expected: Features that rank lower than expected, based on feature-feature correlation, are denoted as the features that fall below the confidence intervals in Figure 6.
